# Physiologically based pharmacokinetic (PBPK) modeling of the role of CYP2D6 polymorphism for metabolic phenotyping with dextromethorphan

**DOI:** 10.3389/fphar.2022.1029073

**Published:** 2022-10-24

**Authors:** Jan Grzegorzewski, Janosch Brandhorst, Matthias König

**Affiliations:** Institute for Theoretical Biology, Institute of Biology, Humboldt University, Berlin, Germany

**Keywords:** dextromethorphan (DXM), CYP2D6, physiologically based pharmacokinetic model (PBPK), pharmacokinetics, pharmacogenomics (PGx), metabolic phenotype

## Abstract

The cytochrome P450 2D6 (CYP2D6) is a key xenobiotic-metabolizing enzyme involved in the clearance of many drugs. Genetic polymorphisms in CYP2D6 contribute to the large inter-individual variability in drug metabolism and could affect metabolic phenotyping of CYP2D6 probe substances such as dextromethorphan (DXM). To study this question, we (i) established an extensive pharmacokinetics dataset for DXM; and (ii) developed and validated a physiologically based pharmacokinetic (PBPK) model of DXM and its metabolites dextrorphan (DXO) and dextrorphan O-glucuronide (DXO-Glu) based on the data. Drug-gene interactions (DGI) were introduced by accounting for changes in CYP2D6 enzyme kinetics depending on activity score (AS), which in combination with AS for individual polymorphisms allowed us to model CYP2D6 gene variants. Variability in CYP3A4 and CYP2D6 activity was modeled based on *in vitro* data from human liver microsomes. Model predictions are in very good agreement with pharmacokinetics data for CYP2D6 polymorphisms, CYP2D6 activity as described by the AS system, and CYP2D6 metabolic phenotypes (UM, EM, IM, PM). The model was applied to investigate the genotype-phenotype association and the role of CYP2D6 polymorphisms for metabolic phenotyping using the urinary cumulative metabolic ratio (UCMR), DXM/(DXO + DXO-Glu). The effect of parameters on UCMR was studied *via* sensitivity analysis. Model predictions indicate very good robustness against the intervention protocol (i.e. application form, dosing amount, dissolution rate, and sampling time) and good robustness against physiological variation. The model is capable of estimating the UCMR dispersion within and across populations depending on activity scores. Moreover, the distribution of UCMR and the risk of genotype-phenotype mismatch could be estimated for populations with known CYP2D6 genotype frequencies. The model can be applied for individual prediction of UCMR and metabolic phenotype based on CYP2D6 genotype. Both, model and database are freely available for reuse.

## 1 Introduction

The cytochrome P450 (CYPs) superfamily of enzymes has a central role in the clearance of many substances and drugs, with the isoform 2D6 (CYP2D6) being one of the most important xenobiotic-metabolizing enzymes. CYP2D6 is involved in the clearance of around 20% of the most prescribed drugs (Saravanakumar et al., 2019) including antiarrhythmics having a small therapeutic range (e.g., flecainide, procainamide, mexiletine), anticancer agents (e.g., tamoxifen), antidepressants (e.g., citalopram, fluoxetine, duloxetine: venlafaxine), antipsychotics (e.g., aripiprazole, haloperidol, thioridazine), *β*-blockers (metoprolol), analgesics (tramadol, oxycodone, codeine), and antitussives (dextromethorphan) (Hurtado et al., 2020; Kibaly et al., 2021). CYP2D6-mediated drug response exhibits a particularly large inter-individual variability which poses a challenge for personalized dosage of medication by underdosing on the one hand and toxic side effects on the other. The activity of CYP2D6 is known to be majorly dependent on genetic variants (Berm et al., 2013; Preskorn et al., 2013; Shah and Smith, 2015) with polymorphism of CYP2D6 being related to the risk of adverse effects, non-response during treatment, and death by drug intoxication (Gasche et al., 2004; Kawanishi et al., 2004; Rau et al., 2004; Zackrisson et al., 2010).

In the late 70 s, a polymorphism in debrisoquine hydroxylation (Mahgoub et al., 1977) and sparteine oxidation (Eichelbaum et al., 1979) was discovered and subsequently attributed to allelic variants of the CYP2D6 gene. In the following years, CYP2D6 became one of the most studied drug-metabolizing enzymes. Genetic variants were classified into distinct phenotypes and subjects carrying combinations of these variants were categorized as poor metabolizer (gPM), intermediate metabolizer (gIM), extensive metabolizer (gEM), and ultra rapid metabolizer (gUM) (Zanger et al., 2004; Gaedigk et al., 2017). This classification is based on the relationship between genetic variants and CYP2D6-mediated drug response. For these genetically predicted phenotypes, we use the “g” nomenclature as they can be easily confused with the actual *in vivo* metabolic phenotype, determined based on pharmacokinetic measurements after the administration of CYP2D6 test drugs. Nowadays, the CYP2D6 activity score (AS) system, a more refined metric, is often applied to characterize genotype-phenotype associations (Gaedigk et al., 2018a). In the system, discrete values between 0 and 1 are assigned to gene variants. The final activity score is calculated by the sum of the activity scores of both alleles. For instance, a person with diplotype *1/*3 (the variant *1 has an AS of 1 and the variant *3 has no activity with an AS of 0) has an overall AS of 1. Higher activity scores than 2 and additional complexity arise from copy number variation (CNV), chimeras, and hybrids with the pseudo gene CYP2D7. This can result in ambiguities and difficulties in the assignment of the correct diplotype and activity score (Gaedigk et al., 2007; Nofziger and Paulmichl, 2018; Gaedigk et al., 2019). Of note, AS specifics are still under heavy debate and regularly updated (Caudle et al., 2020). A multitude of population studies have been conducted to identify and associate allele variants with metabolic phenotypes within and across populations (Gaedigk et al., 2017). Over 130 CYP2D6 star (*) allele haplotypes have been identified and subsequently cataloged by the Pharmacogene Variation (PharmVar) Consortium into PharmGKB with their respective activity score contribution (Gaedigk et al., 2018b; Whirl-Carrillo et al., 2021).

Various methods exist for the metabolic phenotyping based on test substances. The gold standard is plasma concentration sampling of probe substances and their metabolites at various time points after the administration. (Partial) clearance rates and the relative enzyme activities can be calculated from these plasma time profiles. Simplified methods have been established for many probe substances which do not require repeated sampling of blood, e.g., the (cumulative) metabolic ratios between the probe substance and one or several of its metabolites at a single time point in blood, plasma, or urine are utilized as such proxy measures. Large-scale population studies often tend to employ urinary ratios of metabolites. Alternatively, sampling of saliva and breath are worth considering (De Kesel et al., 2016). Probe substances for metabolic phenotyping of CYP2D6 are debrisoquine, dextromethorphan, metoprolol, or sparteine (Frank et al., 2007; Fuhr et al., 2007). Bufuralol is less popular but well suited for *in vitro* investigations due to its fluorescent properties (Zanger et al., 2004). Although debrisoquine and sparteine have excellent properties for CYP2D6 phenotyping, they have been withdrawn from clinical use in most countries and are therefore no longer readily available. Frequently in use for the phenotyping of CYP2D6 activity are metoprolol and dextromethorphan.

Dextromethorphan (DXM) is an over-the-counter, antitussive, non-narcotic, synthetic analog of codeine affecting the activity of numerous channels and receptors in the brain that trigger the cough reflex (Silva and Dinis-Oliveira, 2020). It is generally well-tolerated, considered safe in therapeutic dosage, and easily available (Fuhr et al., 2007). Besides therapeutic purposes, DXM is most commonly applied as a probe substance for CY2D6 phenotyping, alone or with other probe substances in a cocktail. DXM can be administered orally and intravenously, has low bioavailability (≈50%) and a rapid first-pass effect in the intestine and liver. Typically only about half of the dose is recovered in urine over at least 12 h after administration, primarily as glucuronides (Schadel et al., 1995; Capon et al., 1996; Tennezé et al., 1999; Strauch et al., 2009). In the systemic circulation, ≈ 55–65% of DXM is non-specifically bound to plasma proteins (Lutz and Isoherranen, 2012; Taylor et al., 2016).

The biotransformation of DXM is mostly confined to the liver, where DXM is O-demethylated by CYP2D6 to the active metabolite dextrorphan (DXO). Subsequently to O-demethylation, most of the DXO is rapidly transformed *via* UDP-glucuronosyltransferase (UGT) to dextrorphan O-glucuronide (DXO-Glu) and excreted *via* the urine. In individuals without any functional variant of CYP2D6, the metabolization of DXM to DXO is extremely slow but still present. Apparently, the O-demethylation is not exclusively mediated by CYP2D6, and it has been demonstrated *in vitro* that O-demethylation of DXM can be marginally mediated by CYP3A4, CYP3A5 and CYP2C9 (von Moltke et al., 1998; McGinnity et al., 2000; Takashima et al., 2005; Yu and Haining, 2001). In line with this observation, inhibition of CYP2D6, e.g., barely affects poor metabolizer (Pope et al., 2004). The second pathway of DXM metabolization goes *via* N-demethylation to 3-methoxymorphinan which is mainly catalyzed *via* CYP3A4. Subsequently, 3-methoxymorphinan and DXO are biotransformed to 3-hydroxymorphinan which is then rapidly transformed *via* glucuronidation to hydroxymorphian O-glucuronide and excreted in the urine. The urinary cumulative metabolic ratio (UCMR) of DXM to its metabolites DXM/(DXO + DXO-Glu) is a widely applied measure for the *in vivo* CYP2D6 phenotyping.

An crucial question for metabolic phenotyping and liver function testing is how CYP2D6 polymorphisms affect the pharmacokinetics of DXM and metabolic phenotyping based on DXM, such as the UCMR. The objective of this work was to answer this question by the means of physiologically based pharmacokinetic (PBPK) modeling of DXM.

## 2 Material and methods

### 2.1 Pharmacokinetics database of DXM

Pharmacokinetics data of DXM was systematically curated from literature for model development, parameterization, and validation. Curation efforts were mainly focused on concentration-time profiles of DXM, DXO, and DXO-Glu in plasma or serum and their amounts or ratios in urine. The data is accompanied by metadata on the investigated subjects and groups (e.g., CYP2D6 genotype or activity score) and the applied intervention (e.g., dose and application form of DXM). All data was curated using an established curation pipeline (Grzegorzewski et al., 2022) and is available *via* the pharmacokinetics database PK-DB (https://pk-db.com) (Grzegorzewski et al., 2021). As a first step, a PubMed search for the pharmacokinetics of dextromethorphan in combination with genotyping and/or phenotyping was performed with the search query https://pubmed.ncbi.nlm.nih.gov/?term=dextromethorphan+ AND+%28phenotype+OR+phenotyping%29+AND+genotype. The literature corpus was extended with drug cocktail studies from PK-DB (Grzegorzewski et al., 2022), secondary literature from references, and results from PKPDAI with the search query https://app.pkpdai.com/?term=dextromethorphan (Gonzalez Hernandez et al., 2021). Data was selected and curated based on eligibility criteria, see below. During the curation process, the initial corpus was updated by additional publications from the references and citations. A subset of the studies only reported pharmacokinetic parameters without timecourses. These studies were curated but not further used in the following analyses.

To be eligible, studies had to report *in vivo* pharmacokinetics data for adult (age ≥18) humans after administration of DXM or DXM hydrobromide. The application route of DXM was restricted to oral (PO) or intravenous (IV). All application forms (e.g., tablet, capsule, solution) were accepted. No restrictions were imposed on the dosing amount of DXM or coadministrations of other substances. Studies containing coadminstrations that inhibit or induce the pharmacokinetics of DXM were identified during the modeling process and excluded. The relevant outcome measures are concentration-time profiles in plasma, serum, and urine amounts of DXM, DXM metabolites, or metabolic ratios of metabolites such as UCMR. Studies containing pharmacokinetic parameters of DXM and its metabolites (e.g., clearance, half-life, AUC) and (urinary cumulative) metabolic ratios of DXM and its metabolites were included. Data containing timecourses and CYP2D6 genotype information were prioritized. Non-healthy subjects were excluded if the disease is known to affect the pharmacokinetics of DXM or DXM metabolites. Study B from the PhD thesis of Frank (2009) highly deviates from the remaining data and was therefore excluded. Further, Wyen et al. (2008) was identified as a duplicate of Study E from the PhD thesis of Frank (2009) and excluded. The final set of curated studies used in the presented analyses is provided in Table 1.

**TABLE 1:** Clinical studies with pharmacokinetics used for model evaluation.

Reference	PK-DB	PMID	DXM application	Dosing protocol	Description
Abdelrahman et al. (1999)	PKDB00573	10340911	DXM	Oral (syrup): 0.3 mg/kg	Investigation of terbinafine as a CYP2D6 inhibitor *in vivo*
Abduljalil et al. (2010)	PKDB00574	20881950	DXM hydrobromide	Oral (capsule): 30 mg	Assessment of activity levels for CYP2D6*1, CYP2D6*2, and CYP2D6*41 genes by population pharmacokinetics of dextromethorphan
Armani et al. (2017)	PKDB00428	10340911	DXM (in cocktail)	Oral (NR): 30 mg	The antitussive effect of dextromethorphan in relation to CYP2D6 activity
Barnhart. (1980)	PKDB00575	7423506	DXM hydrobromide	Oral (capsule): 30 mg	The urinary excretion of dextromethorphan and three metabolites in dogs and humans
Capon et al. (1996)	PKDB00576	8841152	DXM hydrobromide	Oral (NR): 30 mg	The antitussive effect of dextromethorphan in relation to CYP2D6 activity
Chen et al. (2017)	PKDB00577	28512430	DXM	Oral (tablet): 15 mg + water 300 ml	CYP2D6 phenotyping using urine, plasma, and saliva metabolic ratios to assess the impact of CYP2D6*10 on inter-individual variation in a Chinese population
Chládek et al. (2000)	PKDB00578	11214771	DXM hydrobromide	Oral (syrup): 30 mg	*In vivo* indices of CYP2D6 activity: comparison of dextromethorphan metabolic ratios in 4-h urine and 3-h plasma
Demirbas et al. (1998)	PKDB00579	9840216	DXM hydrobromide	Oral (sustained release tablet): 60 mg	Bioavailability of dextromethorphan (as dextrorphan) from sustained release formulations in the presence of guaifenesin in human volunteers
Dorado et al. (2017)	PKDB00580	28271978	DXM	Oral (NR): 15 mg	Lessons from Cuba for global precision medicine: CYP2D6 genotype is not a robust predictor of CYP2D6 ultrarapid metabolism
Doroshyenko et al. (2013)	PKDB00138	23401474	DXM (in cocktail)	Oral (capsule): 30 mg	Drug metabolism and disposition: the biological fate of chemicals
Duedahl et al. (2005)	PKDB00597	15661445	DXM	Intravenous: 0.5 mg/kg	Intravenous dextromethorphan to human volunteers: relationship between pharmacokinetics and anti-hyperalgesic effect
Dumond et al. (2010)	PKDB00499	20147896	DXM (in cocktail)	Oral (solution): 30 mg	A phenotype-genotype approach to predicting CYP450 and P-glycoprotein drug interactions with the mixed inhibitor/inducer tipranavir/ritonavir
Edwards et al. (2017)	PKDB00496	28808886	DXM (in cocktail)	Oral (capsule): 30 mg	Assessment of pharmacokinetic interactions between obeticholic acid and caffeine, midazolam, warfarin, dextromethorphan, omeprazole, rosuvastatin, and digoxin in phase 1 studies in healthy subjects
Eichhold et al. (1997)	PKDB00596	-	DXM hydrobromide	Oral (syrup): 30 mg	Determination of dextromethorphan and dextrorphan in human plasma by liquid chromatography/tandem mass spectrometry
Eichhold et al. (2007)	PKDB00581	16930908	DXM hydrobromide	Oral (solution): 20 mg	Simultaneous determination of dextromethorphan, dextrorphan, and guaifenesin in human plasma using semi-automated liquid/liquid extraction and gradient liquid chromatography tandem mass spectrometry
Frank (2009)	PKDB00582	-	DXM hydrobromide (in cocktail)	Oral (capsule): 30 mg	Evaluation of pharmacokinetic metrics for phenotyping of the human CYP2D6 enzyme with dextromethorphan
Gaedigk (2013)	PKDB00583	24151800	DXM	Oral (syrup): 0.3 mg/kg	Complexities of CYP2D6 gene analysis and interpretation
Hou et al. (1991)	PKDB00584	2015730	DXM hydrobromide	Oral (capsule): 50 mg	Salivary analysis for determination of dextromethorphan metabolic phenotype
Hu et al. (2011)	PKDB00585	21050887	DXM hydrobromide	Oral (sustained release tablet): 30 mg	Floating matrix dosage form for dextromethorphan hydrobromide based on gas forming technique: *in vitro* and *in vivo* evaluation in healthy volunteers
Jones et al. (1996)	PKDB00586	8873685	DXM hydrobromide	Oral (syrup): 30 mg	Determination of cytochrome P450 3A4/5 activity *in vivo* with dextromethorphan N-demethylation
Köhler et al. (1997)	PKDB00587	9429230	DXM	Oral (syrup): 20 mg	CYP2D6 genotype and phenotyping by determination of dextromethorphan and metabolites in serum of healthy controls and of patients under psychotropic medication
López et al. (2005)	PKDB00588	16249913	DXM hydrobromide	Oral (syrup): 30 mg	CYP2D6 genotype and phenotype determination in a Mexican Mestizo population
Lenuzza et al. (2016)	PKDB00598	25465228	DXM (in cocktail)	Oral (tablet): 18 mg	Safety and pharmacokinetics of the (CIME) Combination of Drugs and Their Metabolites after a single oral dosing in healthy volunteers
Montané Jaime et al. (2013)	PKDB00589	23394389	DXM hydrobromide	Oral (NR): 30 mg + water	Characterization of the CYP2D6 gene locus and metabolic activity in Indo- and Afro-Trinidadians: discovery of novel allelic variants
Myrand et al. (2008)	PKDB00497	18231117	DXM (in cocktail)	Oral (NR): 30 mg	Pharmacokinetics/genotype associations for major cytochrome P450 enzymes in native and first- and third-generation Japanese populations: comparison with Korean, Chinese, and Caucasian populations
Nagai et al. (1996)	PKDB00590	8830977	DXM hydrobromide	Oral (tablet): 30 mg	Pharmacokinetics and polymorphic oxidation of dextromethorphan in a Japanese population
Nakashima et al. (2007)	PKDB00599	17652181	DXM hydrobromide	Oral (tablet): 30 mg	Effect of cinacalcet hydrochloride, a new calcimimetic agent, on the pharmacokinetics of dextromethorphan: *in vitro* and clinical studies
Nyunt et al. (2008)	PKDB00591	18362694	DXM	Oral (tablet): 30 mg	Pharmacokinetic effect of AMD070, an Oral CXCR4 antagonist, on CYP3A4 and CYP2D6 substrates midazolam and dextromethorphan in healthy volunteers
Oh et al. (2012)	PKDB00054	22483397	DXM (in cocktail)	Oral (NR): 2 mg	High-sensitivity liquid chromatography-tandem mass spectrometry for the simultaneous determination of five drugs and their cytochrome P450-specific probe metabolites in human plasma
Pope et al. (2004)	PKDB00592	15342614	DXM	Oral (capsule): 30 mg; 45 mg; 60 mg	Pharmacokinetics of dextromethorphan after single or multiple dosing in combination with quinidine in extensive and poor metabolizers
Qiu et al. (2016)	PKDB00600	27023460	DXM hydrobromide	Oral (tablet): 15 mg	Effects of the Chinese herbal formula “Zuojin Pill” on the pharmacokinetics of dextromethorphan in healthy Chinese volunteers with CYP2D6*10 genotype
Schadel et al. (1995)	PKDB00593	7593709	DXM	Oral (capsule): 30 mg	Pharmacokinetics of dextromethorphan and metabolites in humans: influence of the CYP2D6 phenotype and quinidine inhibition
Schoedel et al. (2012)	PKDB00594	22283559	DXM	Oral (capsule): twice daily for 8 days; 30 mg	Randomized open-label drug-drug interaction trial of dextromethorphan/quinidine and paroxetine in healthy volunteers
Tamminga et al. (2001)	PKDB00498	11829201	DXM hydrobromide	Oral (tablet): 22 mg	The prevalence of CYP2D6 and CYP2C19 genotypes in a population of healthy Dutch volunteers
Yamazaki et al. (2017)	PKDB00494	27273149	DXM (in cocktail)	Oral (NR): 30 mg	Pharmacokinetic Effects of isavuconazole coadministration with the cytochrome P450 enzyme substrates bupropion, repaglinide, caffeine, dextromethorphan, and methadone in healthy subjects
Zawertailo et al. (2010)	PKDB00595	20041473	DXM	Oral (capsule): 3 mg/kg	Effect of metabolic blockade on the psychoactive effects of dextromethorphan

NR: not reported, DXM: dextromethorphan.

For the selection and evaluation of studies from the literature, the PRISMA-ScR guidelines were adopted where applicable (Tricco et al., 2018). The initial corpus contained 404 studies. After screening, application of eligibility criteria, and prioritization, a total of 47 studies were curated (see Supplementary Figure S1). Of these studies, 36 contained data used in the present work (Table 1).

### 2.2 PBPK model of DXM

**FIGURE 1:**
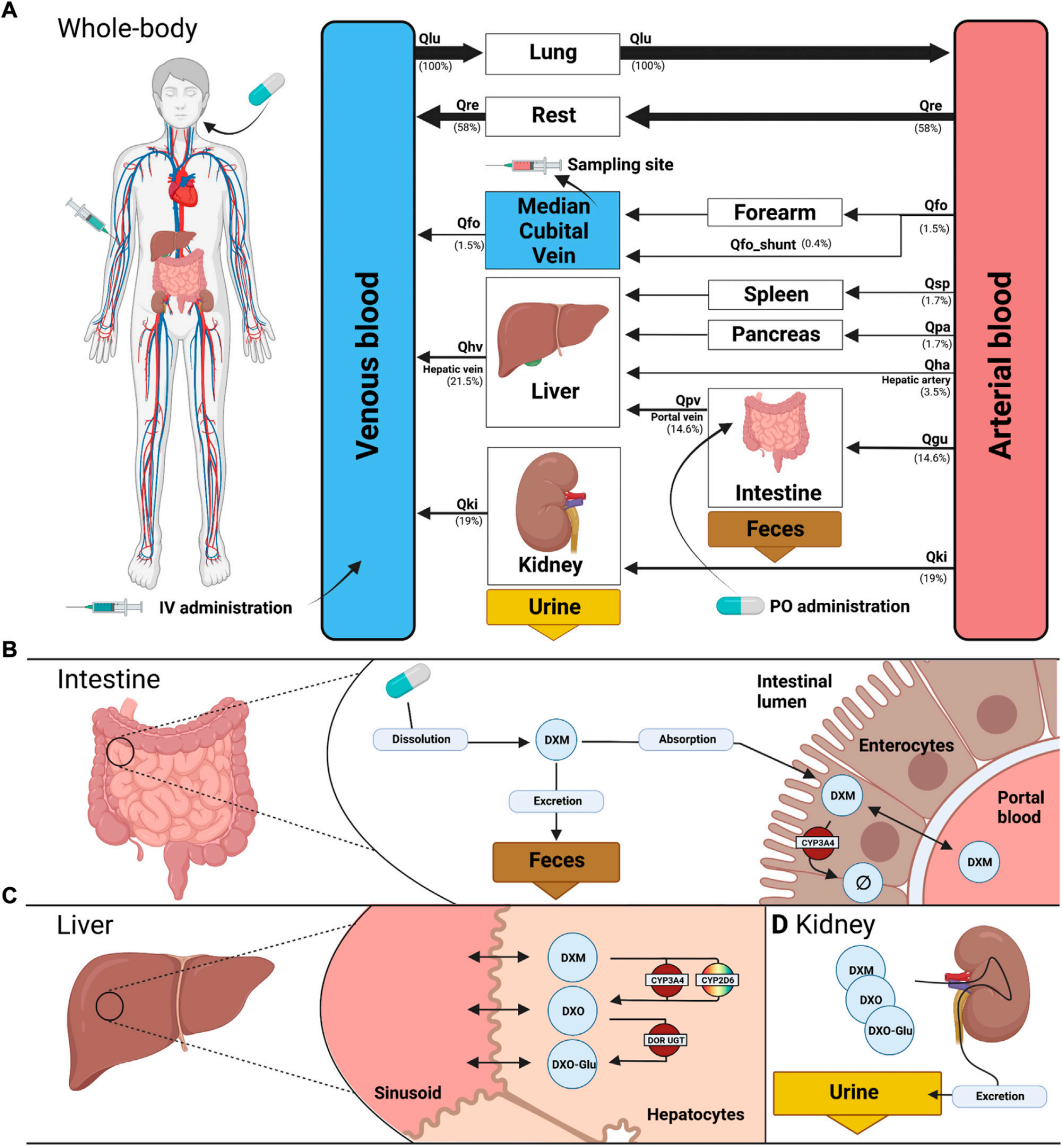
PBPK model of dextromethorphan (DXM). **(A)** whole body model consisting of liver, kidney, intestine, forearm, lung and the rest compartment. Organs with minor relevance are not modeled explicitly and lumped into the rest compartment. Organs are coupled *via* the systemic circulation with arrow width proportional to relative blood flow. DXM can be administered intravenously (IV) or orally (PO) with DXM appearing in the venous blood or intestine, respectively. **(B)** intestine model consisting of dissolution, absorption and excretion of DXM. Only a fraction of DXM is absorbed with the remainder excreted in the feces. First pass metabolism of DXM *via* CYP3A4 in the intestine reduces the amount of DXM appearing in the circulation. **(C)** liver model consisting of DXM → DXO conversion *via* CYP2D6 and CYP3A4 and subsequent glucuronidation to DXO-Glu. **(D)** kidney model for the urinary excretion of DXM, DXO, and DXO-Glu. Created with BioRender.com.

The PBPK model of DXM, DXO, and DXO-Glu (Figure 1) was encoded in the Systems Biology Markup Language (SBML) (Hucka et al., 2019; Keating et al., 2020). For development and visualization, sbmlutils (König, 2021b) and cy3sbml (König et al., 2012; König and Rodriguez, 2019) were used. The model utilizes ordinary differential equations (ODE) which were numerically solved by sbmlsim (König, 2021a) based on the high-performance SBML simulator libroadrunner (Somogyi et al., 2015; Welsh et al., 2022). It is available in SBML under CC-BY 4.0 license from https://github.com/matthiaskoenig/dextromethorphan-model. Within this work, version 0.9.5 of the model was used (Grzegorzewski and König, 2022).

The model is hierarchically organized with submodels coupled using hierarchical model composition (Smith et al., 2015). The top layer represents the whole body with organs and tissues connected *via* the blood flow. The lower layer describes substance-related processes within the tissues. Tissues with minor influence on the pharmacokinetics of DXM, DXO, or DXO-Glu are lumped into the ‘rest’ compartment. Intravenous and oral application of DXM appears in the venous and intestinal compartments, respectively. A fraction of DXM is absorbed *via* the intestinal wall into the systemic circulation. The remainder is excreted *via* the feces. The plasma concentration is evaluated at the median cubital vein.

The distribution of DXM, DXO, and DXO-Glu between plasma and tissue compartments is based on tissue-to-plasma partition coefficients (K_p_) and the corresponding rates of tissue distribution (f_tissue_).

The metabolism of DXM only includes processes relevant for the simulation of the reported pharmacokinetics data (see Figures 1B,C). Routes of minor contribution such as N-demethylation of DXM in the liver were neglected. Metabolic reactions take place in the intestine and liver and are modeled using irreversible Michaelis-Menten reaction kinetics of the form $v=V_{\max}\cdot \frac{S}{S+Km}$ , with V_max_ and K_m_ for CYP3A4 and CYP2D6 sampled from distributions as described below. The conversion of DXM to DXO can be either catalyzed *via* CYP2D6 (main route) or CYP3A4 (minor route) in the liver. Reactions with other products than DXM, DXO, and DXO-Glu were modeled as annihilation, i.e. the products of the reaction are not modeled explicitly. DXM, DXO, and DXO-Glu are eliminated into the urine *via* renal excretion.

A subset of model parameters was fitted by minimizing the distance between model predictions and subsets of the data in Figure 4, Figure 5, Figure 6, Figure 7, Figure 8, and Figure 9.

### 2.3 CYP3A4 and CYP2D6

**FIGURE 2:**
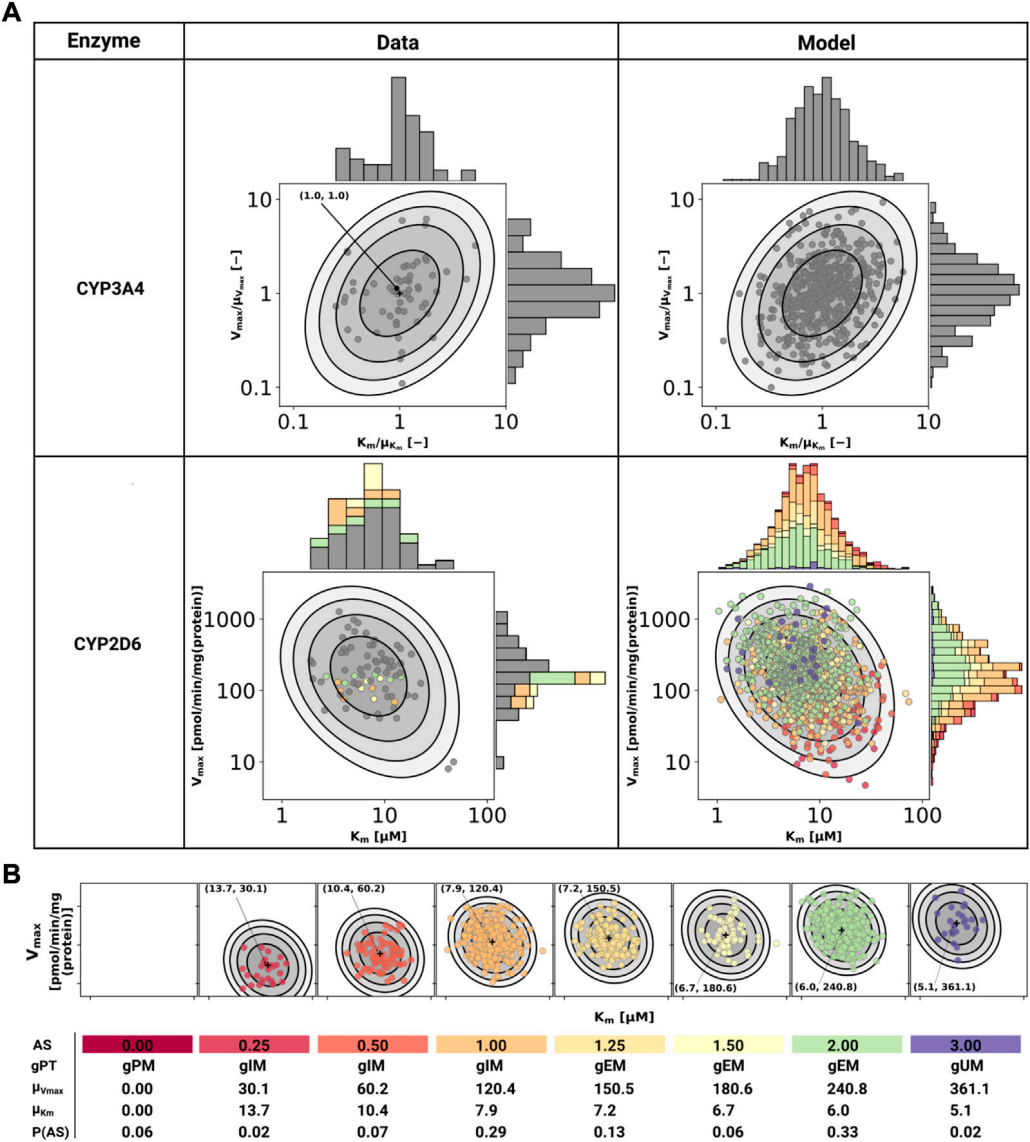
Model of CYP3A4 and CYP2D6. **(A)** CYP3A4 and CYP2D6 distributions. Conversion of DXM → DXO *via* CYP3A4 and CYP2D6 are modeled *via* Michaelis-Menten kinetics. Variability was included *via* two-dimensional lognormal distributions of Michaelis-Menten coefficient (K_m_) and maximum rate of reaction (V_max_). The distribution parameters were determine by fitting to *in vitro* data in human liver microsomes. Variability of CYP3A4 parameters was measured by midazolam (Yang et al., 2012), variability of CYP2D6 parameters *via* DXM (Yang et al., 2012; Storelli et al., 2019a). To transfer the CYP3A4 data from midazolam to DXM normalized values were used. The distribution of CYP2D6 was modeled as a mixture model of the underlying activity scores as depicted in **(B)**. The model CYP3A4 and CYP2D6 distributions were sampled with each point corresponding to a combination of V_max_ and K_m_. CYP2D6 data was color-coded by the respective activity score. **(B)** CYP2D6 activity score model. CYP2D6 activity was modeled *via* a mixture model of individual activity scores. With increasing activity score the V_max_ for the DXM → DXO conversion increases and the μ_Km_ for DXM decreases, i.e., reaction velocity and affinity for the substrate increase. The table provides AS, genetic phenotype (gPT), mean V_max_, mean K_m_, and AS frequency in curated UCMR data (P (AS)). In case of AS = 0.0 CYP2D6 has no activity for the DXM → DXO conversion.

CYP3A4 and CYP2D6 variability was modeled *via* correlated bivariate lognormal distributions fitted to *in vitro* data for CYP2D6 (Yang et al., 2012; Storelli et al., 2019a) and CYP3A4 (Yang et al., 2012), respectively. The data was log10 transformed and a Gaussian, parameterized by the mean (μ) and standard deviation, was fitted by maximum likelihood estimation. The multivariate distribution was realized by a Gaussian copula which in turn was parameterized by Kendall’s tau correlation coefficient from the data (see Figure 2 for data and model).

In order to model the effect of the CYP2D6 AS on the activity, V_max_ was assumed to be proportional to the AS, V_max_ ∝ AS and K_m_ was scaled by the activity score along the first principle component of log10(K_m_) and log10(V_max_) (principal component regression). To model the effect of genetic polymorphisms of CYP2D6, pharmacogenetic variants in the CYP2D6 gene were mapped to their AS and the total activity calculated as the sum of the activity of the two alleles. The genotype-phenotype definitions (i.e. allele variant to AS mapping) were used from PharmGKB (https://www.pharmgkb.org/page/cyp2d6RefMaterials, accessed on 2022-01-10) (Whirl-Carrillo et al., 2021) (Supplementary Table S1).

The stochastic model of CYP2D6 kinetics for a given population consists of a mixture model comprised from the models for each AS weighted by their respective frequency P (AS), i.e., P(V_max_, K_m_) = ∑_AS_P(AS)P(V_max_, K_m_|AS). To simulate a given AS, the respective K_m_ and V_max_ values were used (see Figure 2). The variability in pharmacokinetics was simulated by sampling K_m_ and V_max_ from the CYP3A4 and CYP2D6 distributions. Distributions of CYP3A4 and CYP2D6 parameters were assumed to be statistically independent. To simulate different populations, the AS frequencies for the respective biogeographical population were used from PharmGKB (https://www.pharmgkb.org/page/cyp2d6RefMaterials, accessed on 2022-01-10) (Whirl-Carrillo et al., 2021) (Supplementary Table S2).

### 2.4 CYP2D6 metabolic phenotype

The metabolic phenotypes ultrarapid metabolizer (UM), extensive metabolizer (EM), intermediate metabolizer (IM), and poor metabolizer (PM) were assigned based on the urinary cumulative metabolic ratio of DXM to total dextrorphan $UCMR=\frac{DXM}{DXO+DXO-Glu}$ with the following cutoffs: PM: UCMR ≥0.3, IM: 0.03 ≤ UCMR<0.3, EM: 0.0003 ≤ UCMR<0.03, UM: UCMR<0.0003. Some studies reported the extensive metabolizer as normal metabolizer (NM) with identical cutoffs to the EM. Such data was labeled as EM.

### 2.5 Sensitivity analysis

A local sensitivity analysis of the effect of model parameters on the UCMR was performed. Individual model parameters (p_i_) were varied in both directions by 10% from the base model value $\left(p_{i,-\Delta }\overset{10\%}{\leftarrow }p_{i,0}\overset{10\%}{\to }p_{i,\Delta }\right)$ and the change in the state variable describing the UCMR at 8 h (q) was recorded. The local sensitivity (S (q, p_i_, AS)) was calculated for a range of ASs (0, 0.25, 0.5, 1, 1.25, 1.5, 2.0, 3.0) by the following formula:

$$S\left(q,p_{i},AS\right)=\frac{1}{2}\cdot \frac{q\left(p_{i,\Delta },AS\right)-q\left(p_{i,-\Delta },AS\right)}{p_{i,0}}$$ (1)

## 3 Results

Within this work, a physiologically based pharmacokinetic (PBPK) model of DXM was developed and applied to study the role of the CYP2D6 polymorphism on the pharmacokinetics of DXM and metabolic phenotyping using DXM.

### 3.1 Pharmacokinetics database of DXM

For the development and evaluation of the model, a large pharmacokinetics dataset of DXM and its metabolites, consisting of 36 clinical studies, was established (Table 1). Most of the studies investigated either drug-gene interactions (DGI), drug-drug interactions (DDI), or the interplay of both (i.e. drug-drug-gene interactions). The large majority of studies applied DXM orally (*n* = 35), whereas only a single publication studied DXM pharmacokinetics after intravenous application (*n* = 1) (Duedahl et al., 2005). The application form (i.e., solution, syrup, capsule, table), the used DXM dose (2 mg–3 mg/kg), and coadministrations (i.e., phenotyping cocktail, quinidine, cinacalcet hydrochloride, zuojin) vary between studies, as do sampling times and sampled tissues (i.e., urine, plasma, serum). Importantly, plenty of individual UCMR measurements with corresponding CYP2D6 genotype information are contained within this dataset (*n* = 11 studies). To our knowledge, this is the first large freely available dataset of pharmacokinetics data for DXM with all data accessible from the pharmacokinetics database (PK-DB) (Grzegorzewski et al., 2021).

### 3.2 PBPK model of DXM

Within this work, a PBPK model was developed (Figure 1) to study the role of CYP2D6 polymorphism on DXM pharmacokinetics and metabolic phenotyping with DXM. Important model parameters are provided in Table 2. The model is organized hierarchically, with the top layer representing the whole body (Figure 1A) consisting of the liver, kidney, intestine, forearm, lung, and the rest compartment. Organs with minor relevance are not modeled explicitly and lumped into the rest compartment. Organs are coupled *via* the systemic circulation. DXM can be administered intravenously (IV) or orally (PO) with DXM appearing in the venous blood or intestine, respectively. The intestinal model (Figure 1B) describes dissolution, absorption and excretion of DXM. Only a fraction of DXM is absorbed, with the remainder excreted in the feces. DXM enters the circulatory system by crossing the enterocytes of the intestinal wall. First pass metabolism of DXM *via* CYP3A4 N-demethylation in the intestine reduces the amount of DXM appearing in the systemic circulation. In the liver model (Figure 1C), DXM gets transformed *via* O-demethylation to DXO and subsequently transformed to DXO-Glu. The reactions are modeled by Michaelis Menten kinetics and characterized with K_m_ and V_max_ values. O-demethylation takes place *via* CYP3A4 and CYP2D6. The K_m_ and V_max_ of CYP2D6 is modulated *via* the AS, details can be found in Section 3.3. The kidney model (Figure 1D) describes the urinary excretion of DXM, DXO, and DXO-Glu.

**TABLE 2:** Model parameters in PBPK model of DXM. The complete information is available from the model repository. The prefixes GU__, LI__,KI__, correspond to the intestine/gut, liver, and kidneys, respectively. Values are either adopted from the references or fitted (F). During the robustness analysis of UCMR, various parameters were scanned (S) and a local sensitivity (SA) was performed, see Section 3.5.

Parameter	Description	References	Value	Unit	F	S	SA
BW	Body weight	ICRP (2002) (male)	75	kg			*✓*
HEIGHT	Height	ICRP (2002) (male)	170	cm			*✓*
HR	Heart rate		70	1/min			*✓*
HRrest	Heart rate (resting)		70	1/min			*✓*
COBW	Cardiac output per bodyweight	ICRP (2002); de Simone et al. (1997)	1.548	ml/s/kg		*✓*	*✓*
HCT	Hematocrit	Vander (2001); Herman (2016) (upper range male)	0.51	-			
Kp_fo_dxm	Tissue/plasma partition coefficient DXM forearm		10	-	*✓*		*✓*
f_shunting_forearm	Shunting in forearm		0.2795	-	*✓*		
FVgu	Gut fractional tissue volume	Jones and Rowland-Yeo (2013); ICRP (2002)	0.0171	l/kg			*✓*
FVki	Kidney fractional tissue volume	Jones and Rowland-Yeo (2013); ICRP (2002)	0.0044	l/kg			*✓*
FVli	Liver fractional tissue volume	Jones and Rowland-Yeo (2013); ICRP (2002)	0.021	l/kg		*✓*	*✓*
FVlu	Lung fractional tissue volume	Jones and Rowland-Yeo (2013); ICRP (2002)	0.0076	l/kg			*✓*
FVsp	Spleen fractional tissue volume	Jones and Rowland-Yeo (2013); ICRP (2002)	0.0026	l/kg			*✓*
FVpa	Pancreas fractional tissue volume	Jones and Rowland-Yeo (2013); ICRP (2002)	0.01	l/kg			*✓*
FVfo	Fore arm fractional tissue volume		0.0048	l/kg	*✓*		*✓*
FVve	Venous fractional tissue volume	Jones and Rowland-Yeo (2013); ICRP (2002)	0.0514	l/kg			*✓*
FVar	Arterial fractional tissue volume	Jones and Rowland-Yeo (2013); ICRP (2002)	0.0257	l/kg			*✓*
FVpo	Portal fractional tissue volume	Jones and Rowland-Yeo (2013); ICRP (2002)	0.001	l/kg			*✓*
FQgu	Gut fractional tissue blood flow	Jones and Rowland-Yeo (2013)	0.146	-			*✓*
FQki	Kidney fractional tissue blood flow	Jones and Rowland-Yeo (2013)	0.19	-			*✓*
FQh	Hepatic (venous side) fractional tissue blood flow	Jones and Rowland-Yeo (2013)	0.215	-			
FQlu	Lung fractional tissue blood flow	Jones and Rowland-Yeo (2013)	1	-			*✓*
FQsp	Spleen fractional tissue blood flow	Jones and Rowland-Yeo (2013)	0.017	-			*✓*
FQfo	Fore arm fractional tissue blood flow	RNAO (2022)	0.0146	-			*✓*
FQpa	Pancreas fractional tissue blood flow	ICRP (2002)	0.017	-			*✓*
ftissue_dxm	Vmax tissue distribution DXM		1000	l/min	*✓*		*✓*
Kp_dxm	Tissue/plasma partition coefficient DXM		8.7346	-	*✓*	*✓*	*✓*
Ka_dis_dxm	DXM rate of dissolution and stomach passage		0.0217	1/hr	*✓*		*✓*
Mr_dxo	Molecular weight DXO	CHEBI:29133	257.3707	g/mole			
ftissue_dxo	Vmax tissue distribution DXO		100	l/min	*✓*		*✓*
Kp_dxo	Tissue/plasma partition coefficient DXO		4	-	*✓*		*✓*
Mr_dxo_glu	Molecular weight DXO_glu	CHEBI:32645	433.4948	g/mole			
ftissue_dxo_glu	Vmax tissue distribution DXO_glu		3	l/min	*✓*		*✓*
Kp_dxo_glu	Tissue/plasma partition coefficient DXO_glu		0.08	-	*✓*		*✓*
KI__DXMEX_k	DXM urinary excretion rate		0.017	1/min	*✓*		*✓*
KI__DXOEX_k	DXO urinary excretion rate		0.3	1/min	*✓*		*✓*
KI__DXOGLUEX_k	DXO glucuronide urinary excretion rate		10	1/min	*✓*		*✓*
LI__DXMCYP2D6_Vmax	DXM CYP2D6 Vmax		0.003	mmol/min/l	*✓*		*✓*
LI__DXMCYP2D6_Km	DXM CYP2D6 Km	Storelli et al. (2019a); Yang et al. (2012)	0.0079	mM			*✓*
LI__cyp2d6_ac	CYP2D6 activity score		0.0–3.0	-			*✓*
LI__lambda_1	Slope of Km by principal component regression of (Km, Vmax) in log space	Storelli et al. (2019a); Yang et al. (2012)	-0.4	-	*✓*		
LI__DXMCYP3A4_Vmax	Vmax of DXO formation by CYP3A4		0.0004	mmol/min/l	*✓*		*✓*
LI__DXMCYP3A4_Km	Km of DXO formation by CYP3A4	Yu and Haining (2001)	0.157	mM			*✓*
LI__DXOUGT_Vmax	DXO UGT Vmax (glucuronidation)		0.8953	mmol/min/l	*✓*		*✓*
LI__DXOUGT_Km	DXO UGT Km (glucuronidation)	Lutz and Isoherranen (2012)	0.69	mM			*✓*
GU__F_dxm	Fraction absorbed DXM	Schadel et al. (1995)	0.55	-			*✓*
GU__Ka_abs_dxm	Ka_abs absorption DXM		3.4285	1/hr	*✓*		*✓*
GU__DXMCYP3A4_Vmax	DXM CYP3A4 Vmax		0.0002	mmol/min/l	*✓*		*✓*
GU__DXMCYP3A4_Km	DXM CYP3A4 Km	Kerry et al. (1994); Yu and Haining (2001)	0.7	mM			*✓*
PODOSE	DXM oral dose			mg		*✓*	*✓*

The model allows to predict concentrations and amounts of DXM, DXO, and DXO-Glu depending on CYP2D6 polymorphism, CYP2D6 diplotype, and CYP2D6 AS with amounts and concentrations of DXM, DXO, and DXO-Glu being evaluated in urine or the median cubital vein (plasma).

To our knowledge, this is the first freely accessible, reproducible, and reusable PBPK model of DXM with the model available in SBML from https://github.com/matthiaskoenig/dextromethorphan-model.

### 3.3 CYP3A4 and CYP2D6 variability

Cytochrome P450 enzymes exhibit enormous inter-individual variability in enzyme activity. To account for this variability a stochastic model of CYP2D6 and CYP3A4 activity based on bivariate lognormal distributions of K_m_ and V_max_ was developed and fitted to experimental data from human liver microsomes (Yang et al., 2012; Storelli et al., 2019a) (see Figure 2).

For the CYP2D6 model, the V_max_ is assumed to be linearly related to the AS with *AS* = 0 having no CYP2D6 activity. The dispersion of K_m_ and V_max_ are assumed to be constant for all activity scores. For the mixture model, the frequencies of the individual activity scores P (AS) are adopted from our curated dataset (i.e., relative amount of subjects with reported activity scores and UCMRs). With increasing AS the maximal reaction velocity (V_max_) of DXM conversion *via* CYP2D6 increases as does the affinity for the substrate DXM (K_m_ decreases). The models of CYP3A4 and CYP2D6 are capable of reproducing the data from the literature, but limited information on CYP2D6 genetics within the data hinders the validation of the AS-specific model.

As motivated in the introduction, even subjects carrying no functional variant of the CYP2D6 gene do metabolize DXM to DXO, however extremely slow. This was implemented in the model *via* a secondary O-demethylation *via* CYP3A4 with mean K_m_ for DXM adopted from Yu and Haining (2001). The dispersion of K_m_ and V_max_ is assumed to be identical to the one measured by midazolam in Storelli et al. (2019a) and Yang et al. (2012).

The resulting CYP3A4 and CYP2D6 enzyme model was coupled to the PBPK model and allowed to account (i) for the variability in DXM pharmacokinetics due to the variability in CYPs parameters and (ii) the effect of the AS on CYP2D6 activity and consequently DXM pharmacokinetics.

### 3.4 Effect of CYP2D6 activity score on DXM pharmacokinetics

**FIGURE 3:**
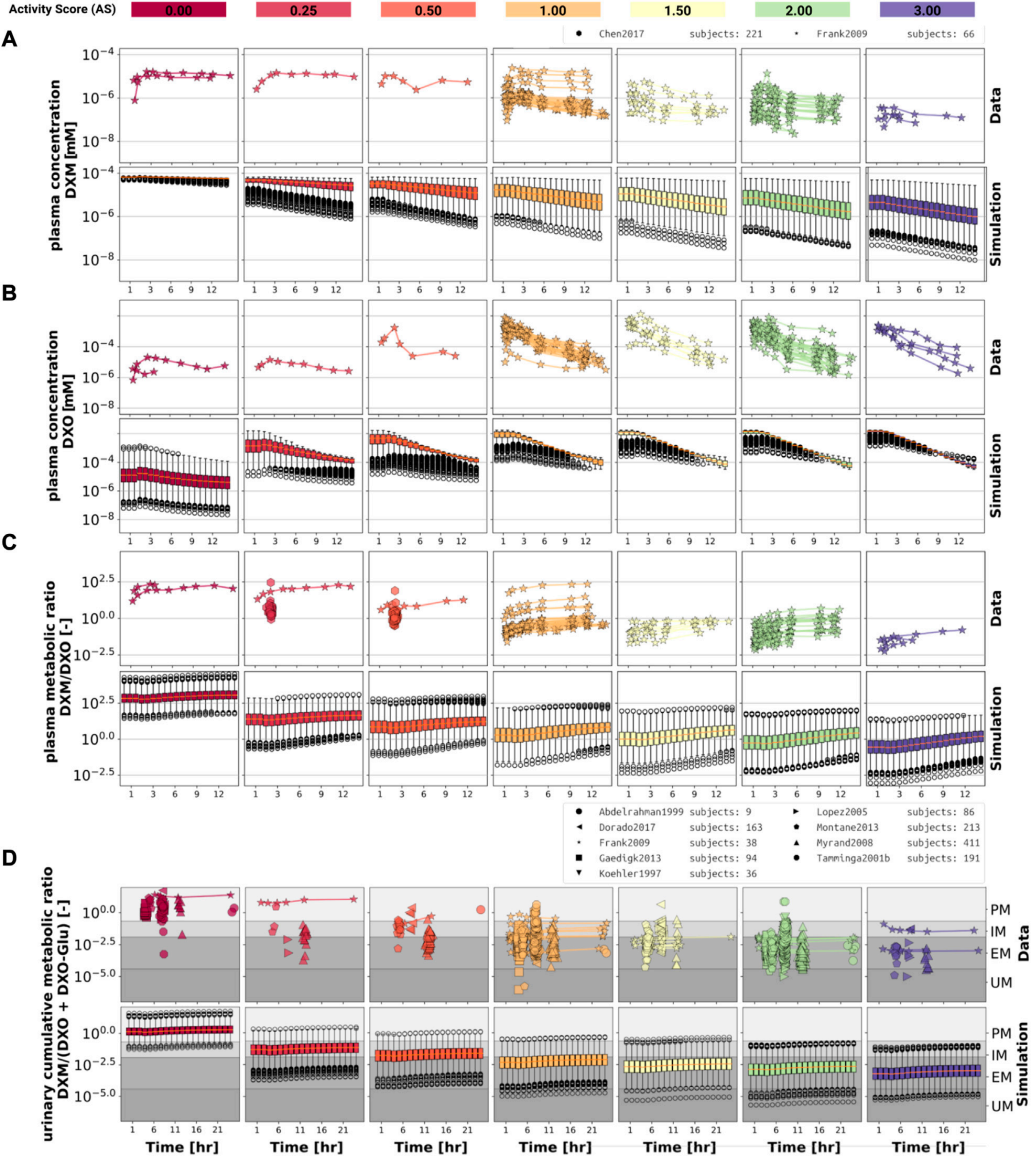
Time-dependency of DXM pharmacokinetics by activity score. **(A)** DXM plasma concentration, **(B)** DXO plasma concentration, **(C)** DXM/DXO plasma ratio, **(D)** UCMR (DXM/(DXO + DXO-Glu) in urine). Depicted is a subset of data in which 30 mg of DXM was applied orally. The upper rows in the panels depict the data in healthy adults from (Köhler et al., 1997; Abdelrahman et al., 1999; Tamminga et al., 2001; López et al., 2005; Myrand et al., 2008; Frank, 2009; Gaedigk, 2013; Montané Jaime et al., 2013; Chen et al., 2017; Dorado et al., 2017). Cocktail studies are included. Studies containing coadminstrations with established drug-drug interactions are excluded. The lower rows depict the respective simulation results. To visualize the large variability in the simulation box plots showing the quartiles along side the median and outliers for selected time points are used. Variables changed in the simulation are the CYP3A4 and CYP2D6 reaction parameters K_m_ and V_max_ according to the distributions in Figure 2. For the different activity scores the respective CYP2D6 activity score model was used.

Model performance was visually assessed for common pharmacokinetic measurements (i.e., DXM, DXO, DXM/DXO in plasma, and DXM/(DXO + DXO-Glu) in urine) and for subjects with reported AS or diplotype (Figure 3). For each AS, a virtual population based on 2,000 K_m_ and V_max_ samples was created from the stochastic models of CYP3A4 and CYP2D6 model. For every AS, an oral application of 30 mg DXM was simulated and compared to the corresponding data. The model predicts large relative variance within a AS group as well as across different AS groups. With increasing AS, and consequently CYP2D6 activity, plasma DXM decreases (Figure 3A), plasma DXO increases (Figure 3B) and the plasma DXM/DXO decreases (Figure 3C) in very good agreement with the data (Frank, 2009; Chen et al., 2017). The large variability within a AS group is a consequence of the large variability of K_m_ and V_max_ in CYP2D6 activity of a single AS (see Figure 2). The large overlap between distributions of adjacent AS results in a large overlap in the pharmacokinetics between neighboring AS.

The UCMR (Figure 3D) is very stable over time with a good agreement with the data. With increasing AS, the UCMR decreases and thereby shifts from PM *via* IM to the EM metabolic phenotype. The UCMR data was pooled independently of the amount of applied DXM (in contrast to A-C only using data from 30 mg oral application) and compared to the simulation as the UCMR endpoint is very robust against the given dose (see Section 3.5).

Overall the model predictions of DXM pharmacokinetics depending on AS are in very good agreement with the available data despite the limited availability of pharmacokinetics timecourses for the low AS 0, 0.25, and 0.5.

**FIGURE 4:**
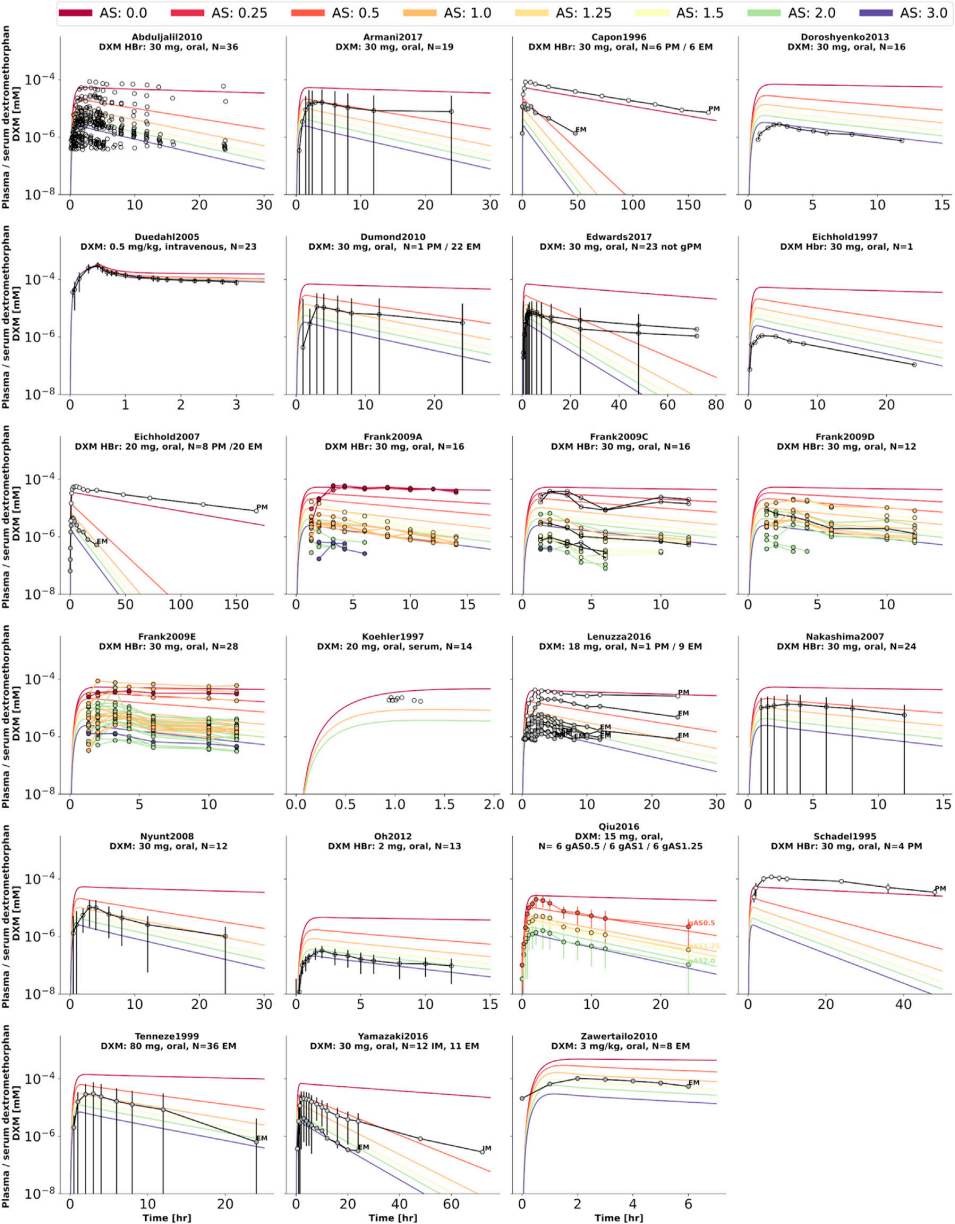
Dextromethorphan (DXM) concentration in plasma or serum. Studies were simulated according to the reported dosing protocol. In case of available activity score information the clinical data is color coded accordingly. Information on metabolizer phenotype (UM, EM, IM, PM) is provided where reported. Data from (Schadel et al., 1995; Capon et al., 1996; Eichhold et al., 1997, 2007; Köhler et al., 1997; Tennezé et al., 1999; Duedahl et al., 2005; Nakashima et al., 2007; Nyunt et al., 2008; Frank, 2009; Abduljalil et al., 2010; Dumond et al., 2010; Zawertailo et al., 2010; Oh et al., 2012; Doroshyenko et al., 2013; Lenuzza et al., 2016; Qiu et al., 2016; Armani et al., 2017; Edwards et al., 2017; Yamazaki et al., 2017).

**FIGURE 5:**
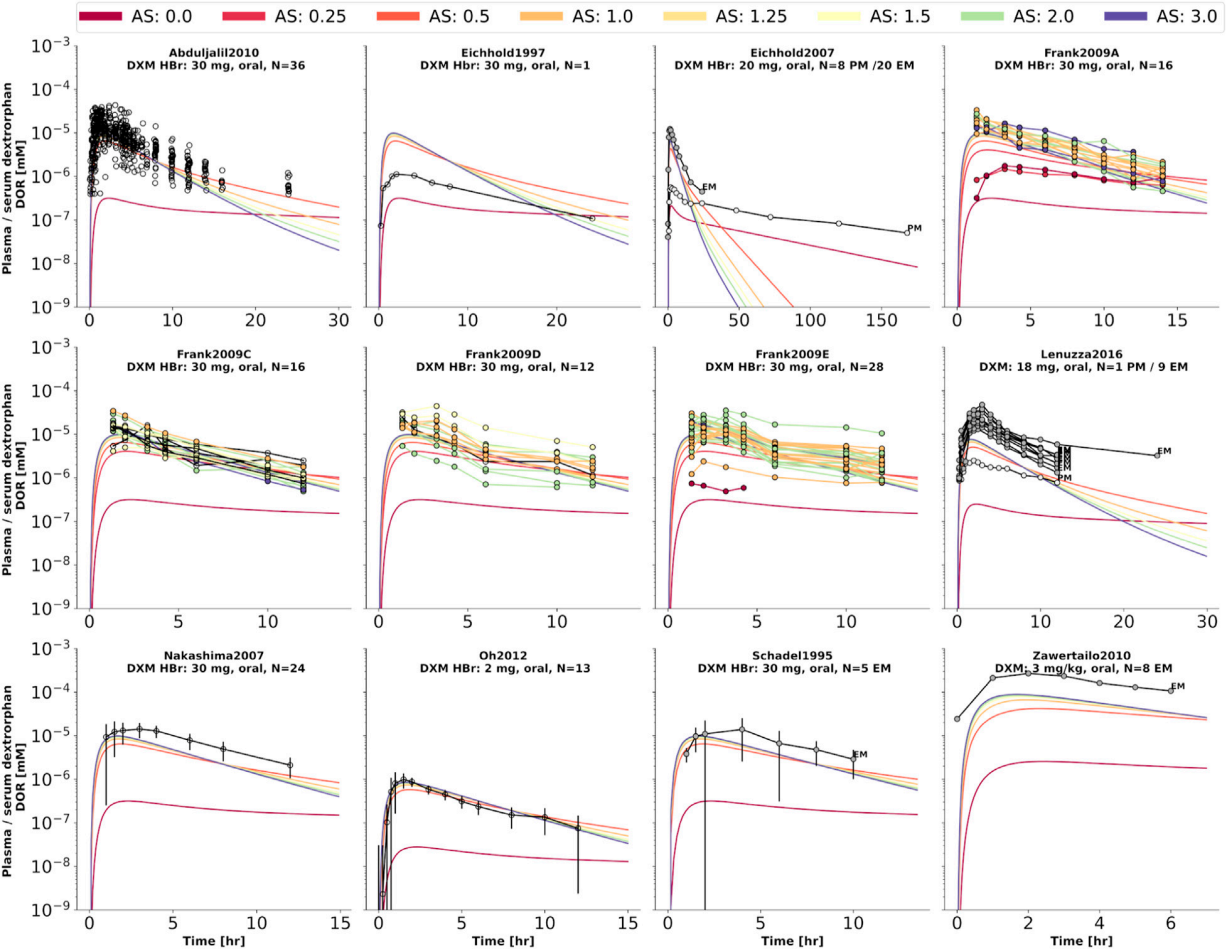
Dextrorphan (DXO) concentration in plasma or serum. Studies were simulated according to the reported dosing protocol. In case of available activity score information the clinical data is color coded accordingly. Information on metabolizer phenotype (UM, EM, IM, PM) is provided where reported. Data from (Schadel et al., 1995; Eichhold et al., 1997, 2007; Nakashima et al., 2007; Frank, 2009; Abduljalil et al., 2010; Zawertailo et al., 2010; Oh et al., 2012; Lenuzza et al., 2016).

**FIGURE 6:**
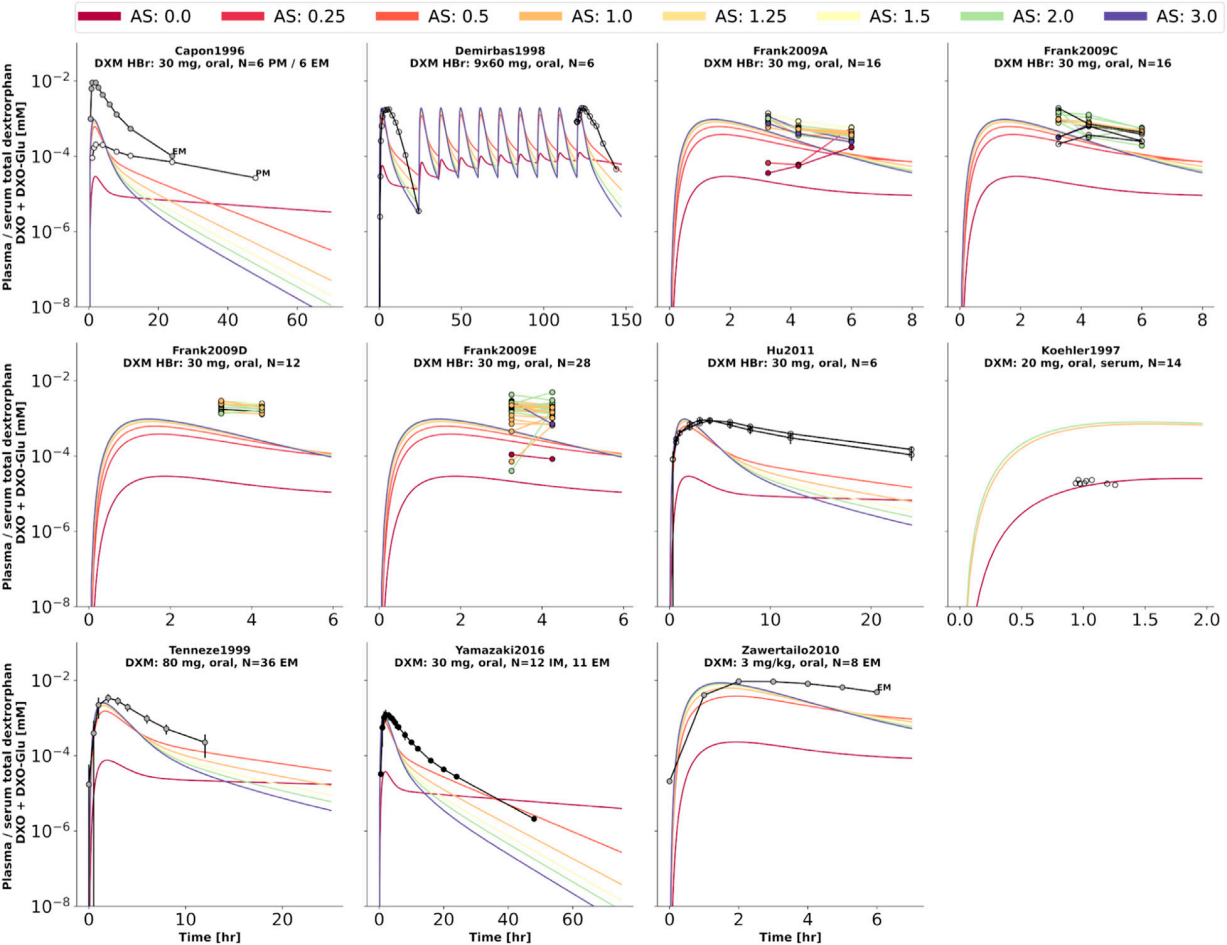
Total dextrorphan (DXO + DXO-Glu) concentration in plasma or serum. Studies were simulated according to the reported dosing protocol. In case of available activity score information the clinical data is color coded accordingly. Information on metabolizer phenotype (UM, EM, IM, PM) is provided where reported. Data from (Capon et al., 1996; Köhler et al., 1997; Demirbas et al., 1998; Tennezé et al., 1999; Zawertailo et al., 2010; Hu et al., 2011; Yamazaki et al., 2017).

**FIGURE 7:**
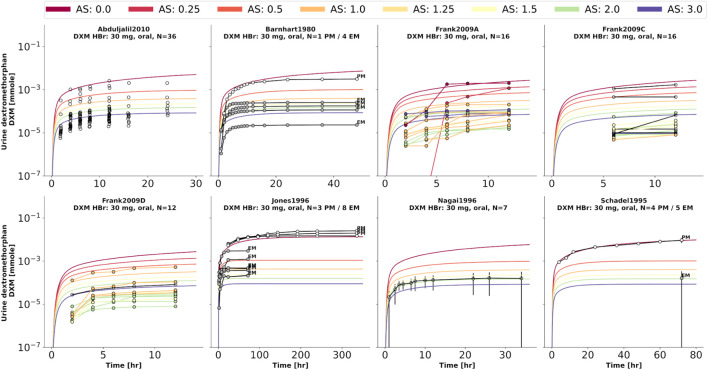
Dextromethorphan (DXM) amount in urine. Studies were simulated according to the reported dosing protocol. In case of available activity score information the clinical data is color coded accordingly. Information on metabolizer phenotype (UM, EM, IM, PM) is provided where reported. Data from (Barnhart, 1980; Schadel et al., 1995; Jones et al., 1996; Nagai et al., 1996; Frank, 2009; Abduljalil et al., 2010).

**FIGURE 8:**
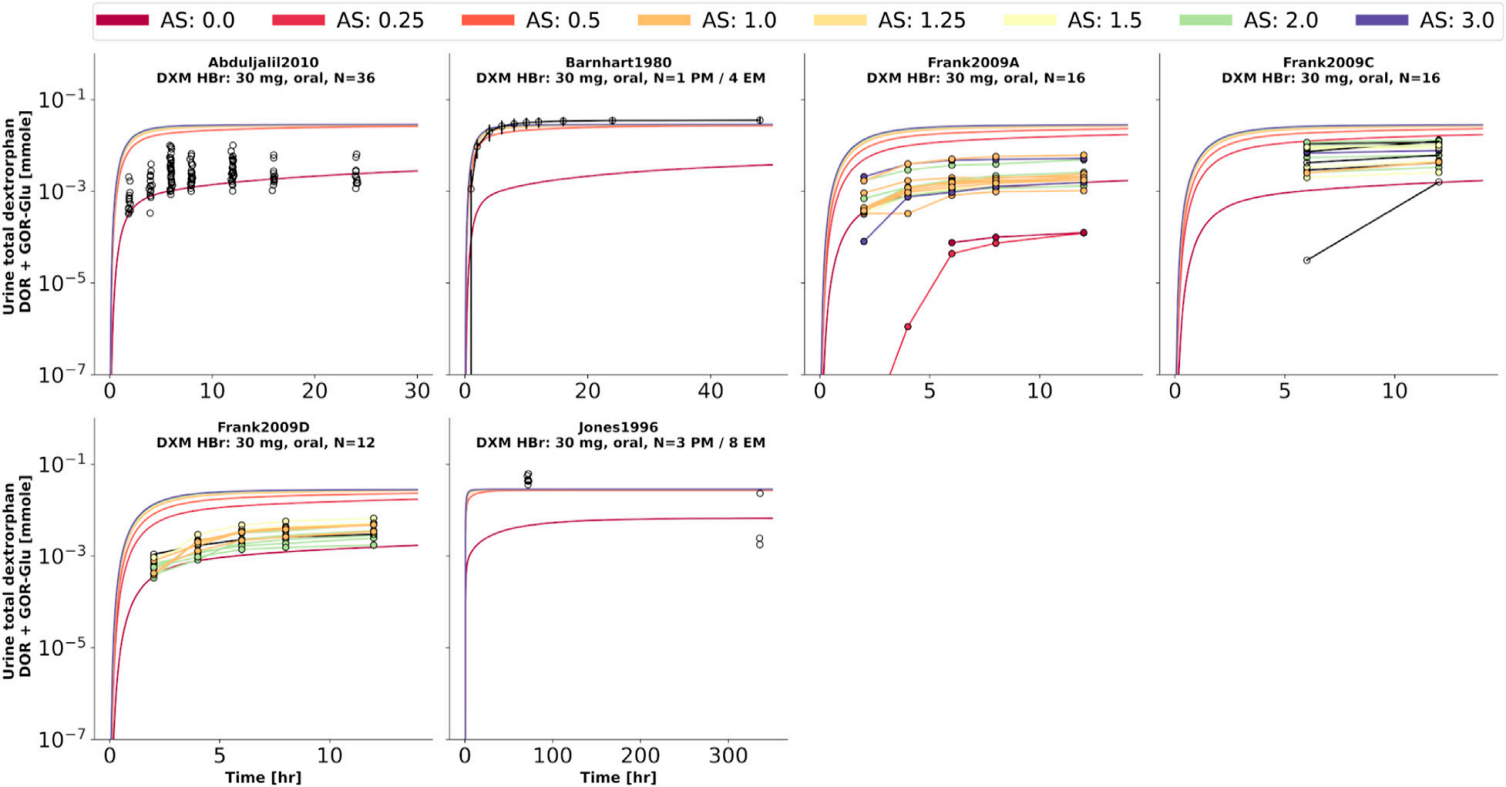
Total dextrorphan (DXO + DXO-Glu) amount in urine. Studies were simulated according to the reported dosing protocol. In case of available activity score information the clinical data is color coded accordingly. Information on metabolizer phenotype (UM, EM, IM, PM) is provided where reported. Data from (Barnhart, 1980; Jones et al., 1996; Frank, 2009; Abduljalil et al., 2010).

**FIGURE 9:**
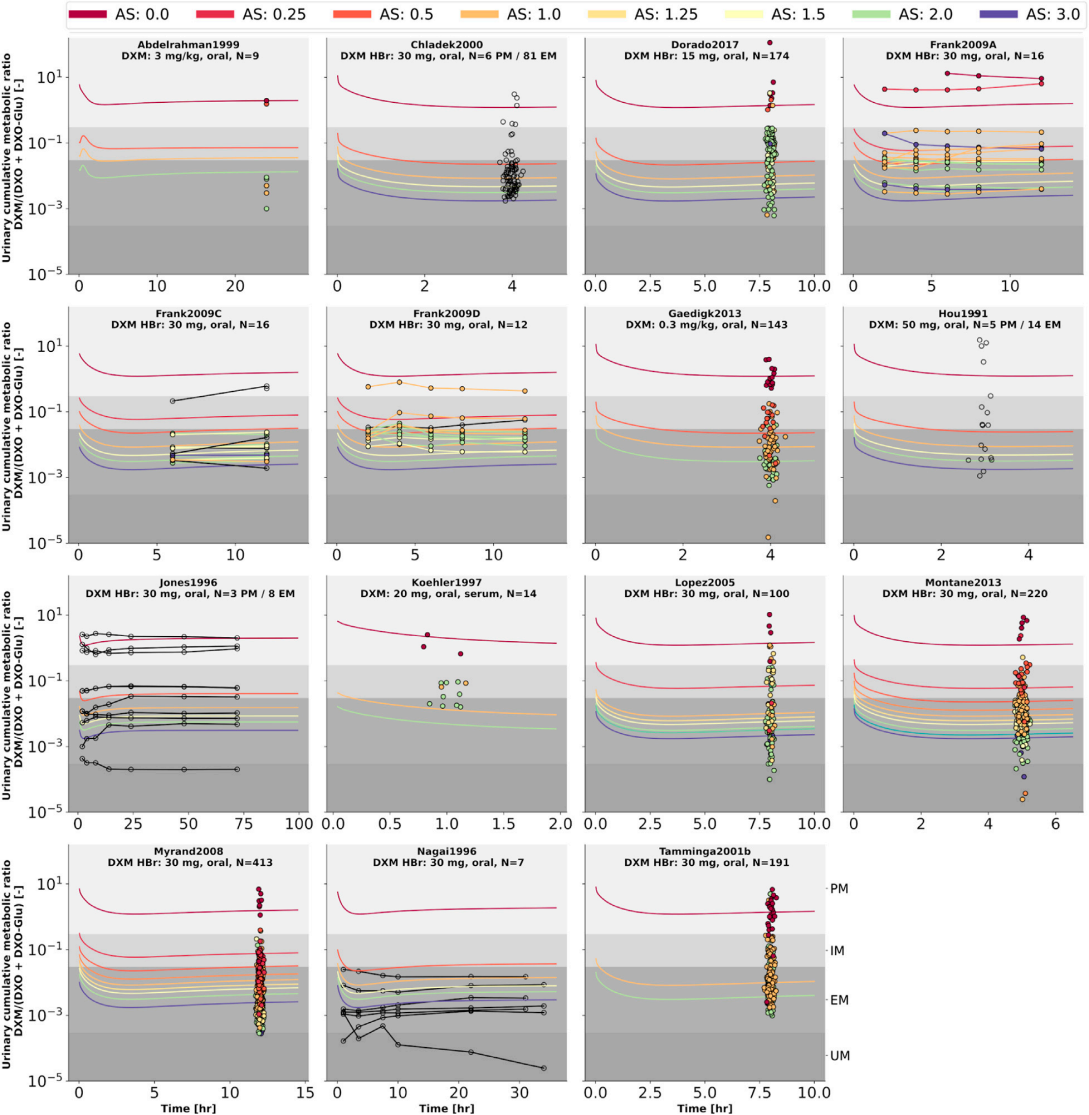
Cumulative metabolic ratio between dextormethorphan and total dextrorphan (DXM/(DXO + DXO-GLU)) in urine (UCMR). Studies were simulated according to the reported dosing protocol. In case of available activity score information the clinical data is color coded accordingly. Information on metabolizer phenotype (UM, EM, IM, PM) is provided where reported. Data from (Hou et al., 1991; Jones et al., 1996; Nagai et al., 1996; Köhler et al., 1997; Abdelrahman et al., 1999; Chládek et al., 2000; Tamminga et al., 2001; López et al., 2005; Myrand et al., 2008; Frank, 2009; Gaedigk, 2013; Montané Jaime et al., 2013; Dorado et al., 2017). The metabolic phenotype definitions for UM, EM, IM, PM are depicted as gray areas.

To further evaluate the model performance, simulations were compared to pharmacokinetics data for DXM in plasma or serum (Figure 4), DXO in plasma or serum (Figure 5), and DXO + DXO-Glu in plasma or serum (Figure 6), DXM in urine (Figure 7), DXO + DXO-Glu in urine (Figure 8), and the UCMR (Figure 9). With expected variability in mind, the model is capable to reproduce all data from the pharmacokinetics dataset. Minor shortcomings of the model are faster kinetics of DXO + DXO-Glu in plasma (Figure 6).

### 3.5 Effect of parameters on metabolic phenotyping *via* UCMR

Analysis of the effect of parameter changes on UCMR is highly relevant as it can help to identify potential confounding factors and bias in UCMR based phenotyping. Of special importance is the question if there is a dependency on the genetic polymorphism (activity score) of these effects.

**FIGURE 10:**
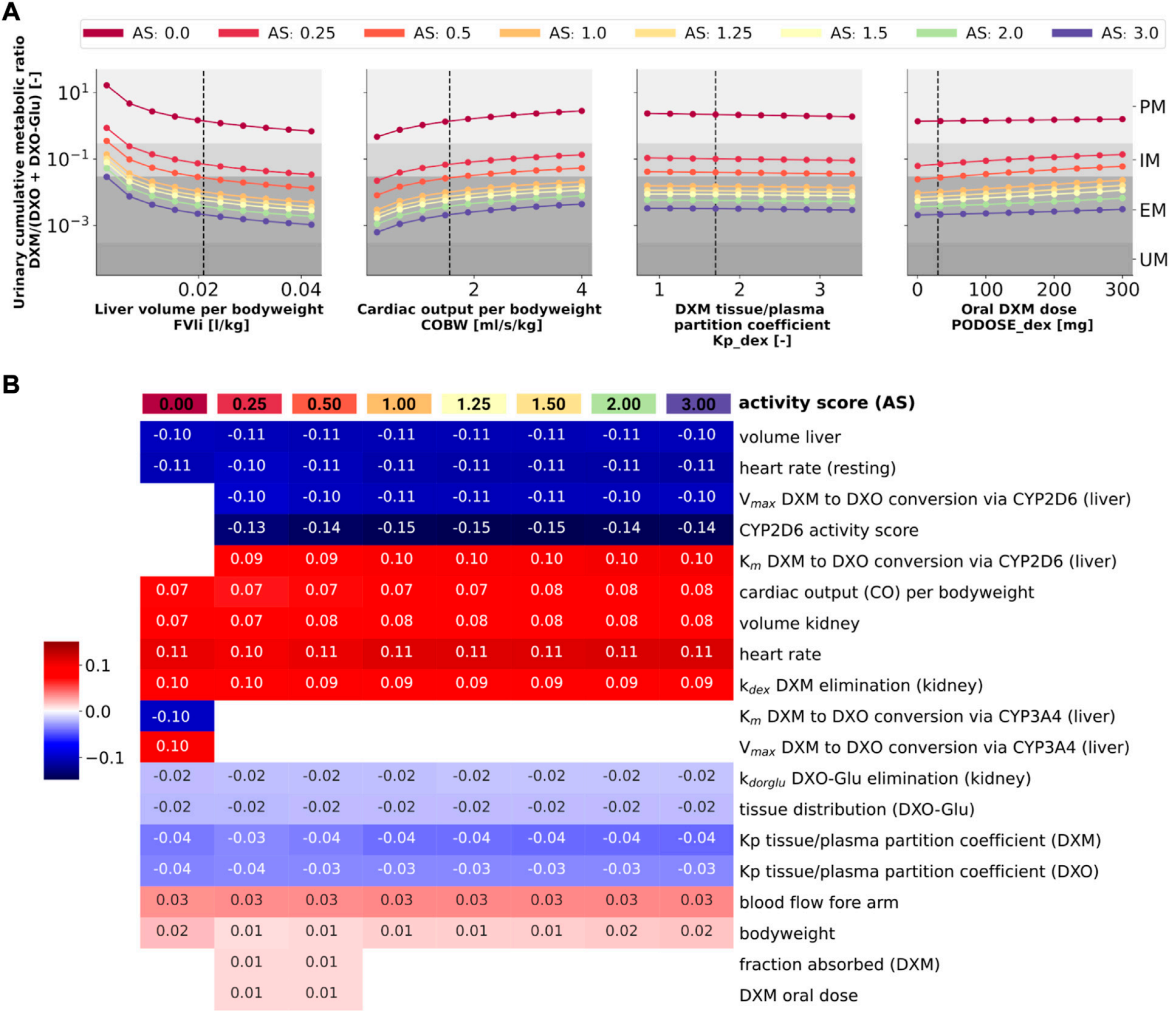
Sensitivity analysis of UCMR by activity score. **(A)** Dependency of UCMR (urinary cumulative ratio of DXM/(DXO-Glu) after 8 h and 30 mg oral DXM) on selected physiological parameters and the DXM dose. Parameter scans were performed for all activity scores. Reference model parameters are depicted as dashed lines. **(B)** Sensitivity analysis of model parameters. To systematically study the effect of parameter changes the local sensitivity of UCMR were calculated for all activity scores. Parameters were varied 10% in both direction around the reference parameter value and the relative change of UCMR was calculated (insensitive parameters with relative change of UCMR smaller than 1% were omitted). Positive sensitivities are depicted in red, negative sensitivities in blue. Parameters were sorted *via* agglomerative clustering. Representative parameters of the clusters (i.e., liver volume per bodyweight, cardiac output per bodyweight, DXM tissue/plasma partition coefficient, and oral DXM dose) are depicted in A. The local sensitivity for the activity scores in B corresponds to the normalized slope at the dashed lines in A.

To answer this question, model parameters (i.e., liver volume, cardiac output, tissue-to-plasma partition coefficient of DXM, and oral dose) were changed in reasonable ranges and the effect on UCMR at 8 h after the application of 30 mg of DXM was investigated (Figure 10A). Independent of the AS, UCMR increased with increasing liver volume and decreased with increasing cardiac output. A change in the tissue-to-plasma partition coefficient of DXM or the amount of oral DXM barely affected the UCMR.

CYP2D6 phenotyping by UCMR is very stable over time as demonstrated in the time course predictions (see 3D and Figure 9) and robust against changes in factors related to the intervention protocol (i.e. dosing amount of DXM, dissolution rate) and to some extent against changes in physiological parameters (see local sensitivity analysis of UCMR in Figure 10B).

Liver volume, heart rate, cardiac output, kidney volume, and kidney elimination rate of DXM altered the UCMR with a similar magnitude as the CYP2D6 reaction parameters. However, the biological variation in these physiological parameters is orders of magnitude lower. The sensitivity analysis showed no effect of UGT *V*_max_ and *K*_*m*_ on UCMR which is the reason why inter-individual variability of UGT activity was not further investigated in this work. Local sensitivity of UCMR was almost identical at different AS values for almost all parameters, i.e., the effect of physiological parameters is of similar relative magnitude independent of AS. For AS = 0, our model assumptions of minor DXM metabolism by CYP3A4 lead to UCMR not being modulated by CYP2D6 but rather by CYP3A4 activity. Nonetheless, even across studies with non-standardized intervention protocols the UCMR measurements seam to be a good but not perfect endpoint to quantify and compare CYP2D6 enzyme activity. Importantly, our analysis indicates that UCMR measurements can be pooled even across investigations with different intervention protocols (as for instance performed in Figure 3D). This still may lead to biases and errors, e.g., due to differences in the quantification protocol.

### 3.6 Effect of CYP2D6 polymorphisms and activity score on UCMR

**FIGURE 11:**
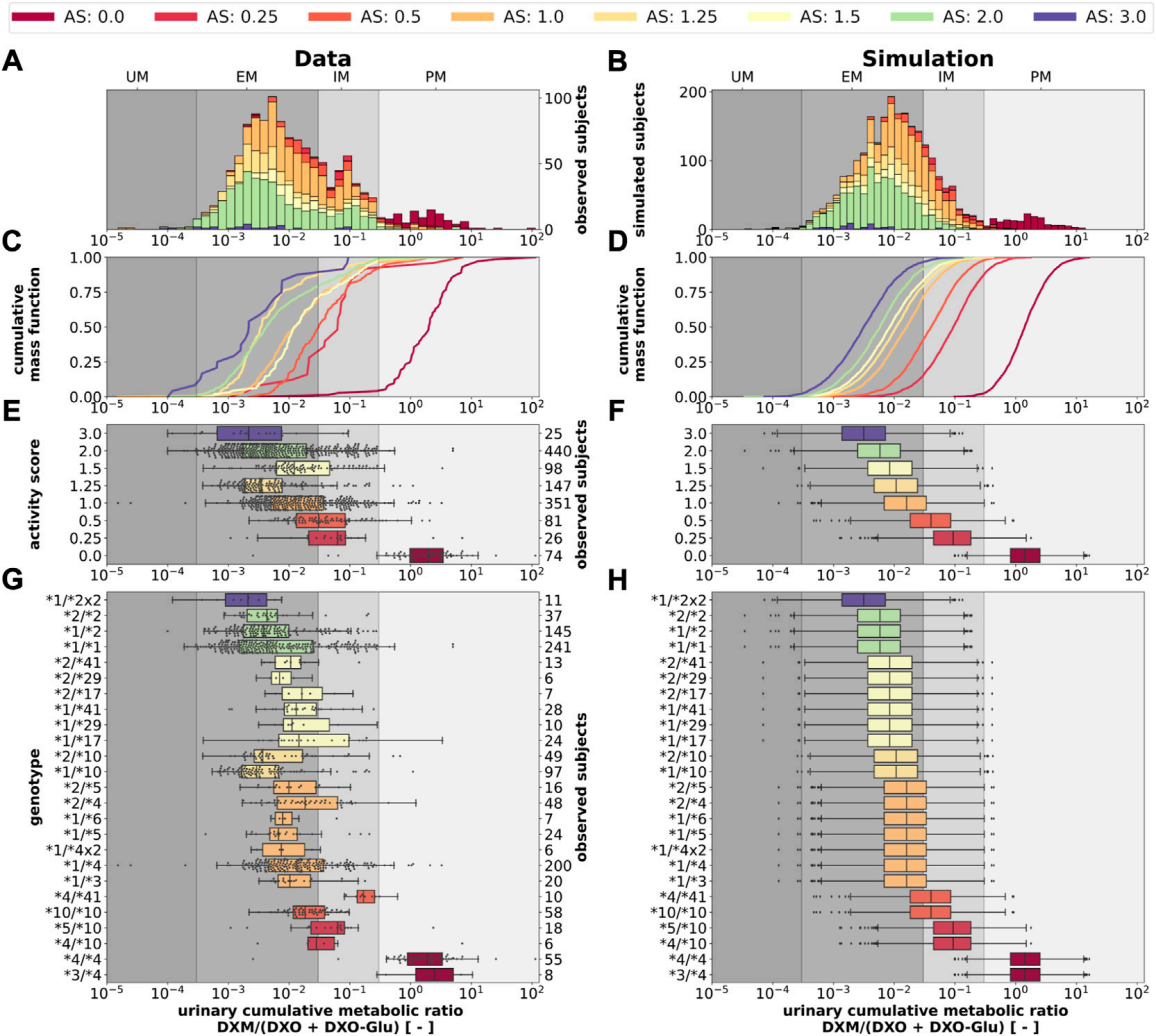
CYP2D6 genotype-, activity score association of the UCMR. Simulation of urinary cumulative ratio of DXM/(DXO-Glu) (UCMR) based on activity score frequencies. UCMR data was measured at least 4 h after the application of DXM (hydrobromide) in healthy adults. Cocktail studies were included in the analysis. Studies containing coadminstrations with established drug-drug interactions were excluded. The ranges for metabolic phenotypes (UM, EM, IM, PM) are depicted as gray shaded areas. For timecourse UCMRs, only the latest measurement after administration was included. Data from (Köhler et al., 1997; Abdelrahman et al., 1999; Tamminga et al., 2001; López et al., 2005; Myrand et al., 2008; Frank, 2009; Gaedigk, 2013; Montané Jaime et al., 2013; Dorado et al., 2017). **(A)** Histogram of UCMR data stratified by CYP2D6 activity score. **(B)** Corresponding simulation results (UCMR at 8 h) from the Monte Carlo simulation with random variables being the enzyme reaction parameter (i.e., K_m_, V_max_). See details in Figure 2. **(C)** Empirical CMFs stratified by the activity scores. **(D)** Corresponding simulated CMFs stratified by CYP2D6 activity scores. **(E)** Box plots of observed UCMRs stratified by CYP2D6 activity scores. **(F)** Box plots of simulated UCMRs stratified by the activity scores. **(G)** Box plots of observed UCMRs stratified by CYP2D6 diplotypes. **(H)** Box plots of simulated UCMRs stratified by CYP2D6 diplotypes. For D, F, and H, 2,000 samples were simulated for each activity score whereas in B and D a two-fold oversampling with the CYP2D6 activity score frequencies from the UCMR data was performed.

Next, we tested if the model is able to predict UCMR distributions for given genotypes and AS (Figure 11). Model predictions based on underlying genotype frequencies were compared with the experimental data. UCMR distributions for individual AS groups are well described by the model. The AS impacts the UCMR, with increasing AS resulting in an decrease in UCMR. However, individual AS distributions heavily overlap, as expected, due to the large overlap in CYP2D6 parameter distributions between different AS. The predicted distributions tend to be slightly narrower than the actual data. Possible reasons are many fold (e.g., omitted physiological variation, omitted variation in UGT activity, difficulties in correct genotype assignment, unknown effect modifiers, and biases).

The AS system could be refined to better describe the data. The categorization of CYP2D6 genotypes into discrete activity values (i. e., 0, 0.25, 0.5, 1) is an oversimplification, a continuous activity score would probably perform better. The model and data indicate that gUM (*AS* ≥ 3) is a very unreliable predictor for ultra rapid metabolism and only gPMs (*AS* = 0) are almost perfectly distinguishable from other metabolizers, see Figures 11C–F.

Another strength of the presented model is that it enables the prediction of the *in vivo* phenotype of subjects based on *in vitro* data.

### 3.7 Population variability in UCMR

**FIGURE 12:**
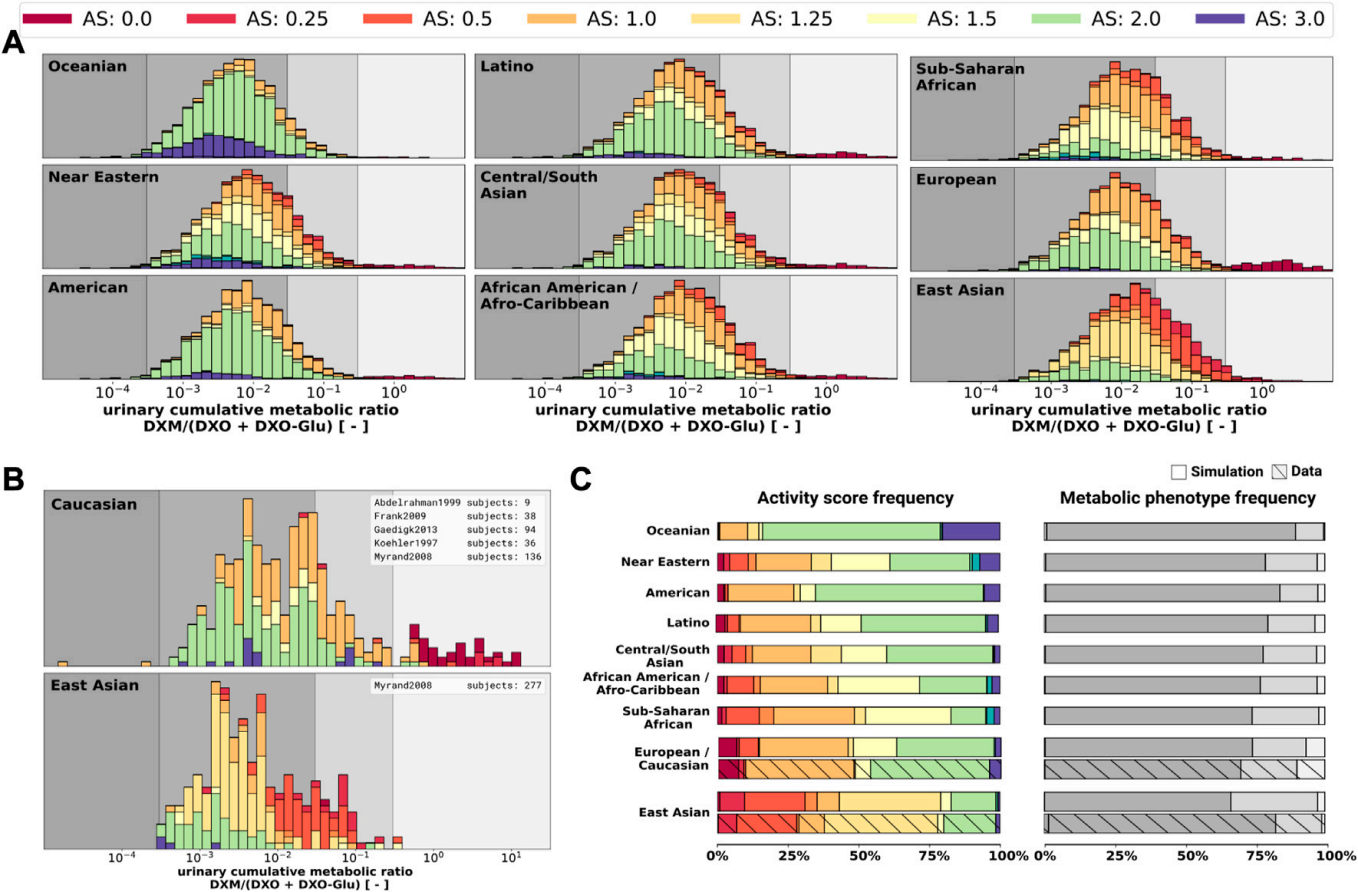
UCMR distributions for biogeographical populations **(A)** Simulated UCMR distributions at 8 h for various biogeographical populations based on reported CYP2D6 activity score frequencies as reported in PharmGKB (Whirl-Carrillo et al., 2021). Frequencies are provided in the Supplementary Table S2. **(B)** Reported UCMRs depending on activity score for Caucasians and East Asians from (Köhler et al., 1997; Abdelrahman et al., 1999; Myrand et al., 2008; Frank, 2009; Gaedigk, 2013). Cocktail studies were included in the analysis. Studies containing coadminstrations with established drug-drug interactions were excluded. **(C)** Simulated activity score frequency and metabolic phenotype frequency for the populations and comparison with data for Caucasian and East Asian populations (hatched bars).

Finally, the model was also capable to predict UCMR distributions for different biogeographical populations (Figure 12) based on the underlying AS frequencies (Supplementary Table S2). Based on the reported frequencies, the UCMR distributions were simulated at 8 h after the application of 30 mg DXM for Oceanian, Near Eastern, American, Latino, Central/South Asian, African American/Afro-Caribbean, Sub-Saharan African, European, and East Asian populations (Figure 12A). Data for Caucasian and East Asian populations (Figure 12B) was used for validation of the predictions (Figure 12C). The data is in good agreement with measurements of Caucasians and East Asians as reported by Abdelrahman et al. (1999); Frank (2009); Gaedigk (2013); Köhler et al. (1997); Myrand et al. (2008).

## 4 Discussion

During the last 20 years various modeling approaches and software solutions were utilized to investigate various aspects of DXM pharmacokinetics, e.g., using GastroPlus (Bolger et al., 2019), P-Pharm (Moghadamnia et al., 2003), SAS (Ito et al., 2010; Chiba et al., 2012), SimCYP (Dickinson et al., 2007; Ke et al., 2013; Sager et al., 2014; Chen et al., 2016; Rougée et al., 2016; Adiwidjaja et al., 2018; Storelli et al., 2019b; Machavaram et al., 2019), MATLAB (Kim et al., 2017), or PK-Sim (Rüdesheim et al., 2022). However, most of the work is difficult/impossible to validate or to build up on due to a lack of accessibility of models and software, and platform-dependency of the models. Here, we provide an openly accessible, reproducible and platform-independent whole-body model of DXM metabolism, which facilitates reusability, extensibility, and comparability.

Apart from that, modeling work which aims for high empirical evidence relies on trustworthy supporting real world data. More and independent sources of data are highly beneficial for the scientific outcomes. For that matter, guidelines like PRISMA for reporting transparency, completeness, and accuracy find very broad endorsement in the field of systematic reviews and meta analysis. The present work faces somewhat similar challenges for the evaluation and selection of data from literature. Therefore, PRISMA-ScR guidelines were adopted where applicable. With this approach, bias within the used dataset could be mitigated or at least identified. Importantly, we supplement our open and accessible model with a large, open, and accessible database of pharmacokinetics data.

The presented PBPK model is able to predict the DXM metabolism of populations and individuals based on their CYP2D6 genotype. It is probably the first model capable to predict individual UCMRs and the expected distributions of UCMR. Moreover, it can reproduce a broad range of reported clinical data on DXM and enables better intuition on how to interpret DXM related pharmacokinetics. E.g., an important message is that CYP2D6 activity is not the only modulator of UCMR, as can be seen by the large variability in activity score and overlap between activity scores. UCMR as a proxy of CYP2D6 metabolic phenotype should therefore be interpreted carefully. The model shows that for extremely low CYP2D6 activity the UCMR is not primarily governed by the CYP2D6 activity. This is consistent with the finding that CYP2D6 inhibition merely affects PMs (Pope et al., 2004).

The current version of the model is already very valuable, still there is plenty of room for improvement. By providing the data and model in open and standardized formats we enable and encourage these improvements by model extensions and updates.

Many of the physiological parameters in the model were fitted or estimated although they could be measured in principle. E.g., relatively low DXM concentrations in plasma suggest substantial extra-vascular binding of DXM. However, tissue-plasma partition coefficients (Kp) are difficult to assess and only limited data is available. Steinberg et al. (1996) reported brain levels to be 68-fold higher and cerebrospinal fluid levels 4-fold lower than serum levels, respectively. Others estimated Kp ∼ 1.65 from n-octanol-water partition coefficients and again others suggested additional trapping mechanisms (i.e. lysosomal trapping) (Bolger et al., 2019). In the model, the DXO-Glu kinetics is a bit to rapid (see Figure 6), probably due to the decision to model tissue distribution uniformly for all organs (i.e., identical K_p_ and f_tissue_). We decided for a more parsimonious model. Glucuronides, however, are generally much more polar than their respective non-glucuronides which result in less plasma binding, higher urinary excretion, lower lipid-solubility, and higher water-solubility. Transport into different tissues is affected differently by polarity.

Most important for model improvements would be additional *in vitro* measurements on the association between CYP2D6 genotype and phenotype which are very limited in literature (Storelli et al., 2019a; Ning et al., 2019; Dalton et al., 2020). Furthermore, simultaneous *in vitro* and UCMR measurements do not exist the literature. Both would be very important for the validation of the AS system and the development of new models which e.g. take into account structural variation (Dalton et al., 2020). For instance, with the AS system alone it is not possible to explain why CYP2D6 is inhibited differently for different genotypes Qiu et al. (2016).

In conclusion, we developed and validated a PBPK model of DXM and applied it to study the effect of the CYP2D6 polymorphism on metabolic phenotyping.

## Data Availability

The original contributions presented in the study are included in the article/Supplementary Material, further inquiries can be directed to the corresponding author. All clinical data of dextromethorphan pharmacokinetics that was used in this work can be found in PK-DB available from https://pk-db.com.
